# Exosomes as Natural Nanocarriers for RNA-Based Therapy and Prophylaxis

**DOI:** 10.3390/nano12030524

**Published:** 2022-02-02

**Authors:** Andrey Gorshkov, Lada Purvinsh, Alexandra Brodskaia, Andrey Vasin

**Affiliations:** 1 Smorodintsev Research Institute of Influenza, Ministry of Health of the Russian Federation, 197376 Saint Petersburg, Russia; ladapurvinsh13@gmail.com (L.P.); a.brodskaya1988@gmail.com (A.B.); vasin_av@spbstu.ru (A.V.); 2 Laboratory of Pathomorphology, Almazov National Research Centre, 197341 Saint Petersburg, Russia; 3 Institute of Biomedical Systems and Botechnologies, Peter the Great St. Petersburg Polytechnic University, 194021 Saint Petersburg, Russia; 4 Scientific and Educational Center for Biophysical Research in The Field of Pharmaceuticals, Saint Petersburg State Chemical Pharmaceutical University, 197022 Saint Petersburg, Russia

**Keywords:** exosomes, nanoparticles, siRNA, miRNA, RNA-based therapy, RNA-binding proteins, targeted delivery

## Abstract

Exosomes are natural nanocontainers actively secreted by the body’s cells and transmitting molecular signals of various types to recipient cells. Cellular mechanisms of exosomes’ biogenesis involve specific sorting of RNA for incorporation into them. As a result, the molecular composition of exosomes is closely related to the donor cell’s functional state, and this makes exosomes an important diagnostic and prognostic marker in a number of diseases (primarily oncological). The ability of exosomes to transport biologically active molecules and to protect the cargo from degradation makes them nearly ideal candidates as delivery carriers of RNA in therapeutic or prophylactic regimes. Potential of exosomal surface functionalization enables improved targeting to specific organs, tissues and cells. However, the development of an effective technology for RNA’s loading into exosomes cannot be considered resolved. This review is focused on experimental data on the use of exosomes as vehicles for the delivery of therapeutic and prophylactic RNAs. We briefly consider the biogenesis and functions of exosomes, focusing on those biological properties that make them formidable candidates in the race to develop effective delivery carriers. Furthermore, we describe various techniques of cargo loading into exosomes. Prospects of exosomes application as therapeutic delivery system for siRNAs, miRNAs, and long RNAs are considered.

## 1. Introduction

RNA-based therapeutics and vaccines are rapidly progressing areas of medical science and making their first steps towards clinical implementation. Agents with potential medical applications include both short and long RNAs. Therapeutic short RNAs include siRNAs, miRNA mimics, and antisense miRNA antagonists [1,2]. Potentially therapeutic long RNAs include mRNAs, long non-coding RNAs, and circular RNAs [3,4,5].

Messenger RNA vaccines have been developed recently as a new tool for immunotherapy for oncological diseases [6,7]. They have also been developed as means of infection prevention, with a number of advantages over classical protein vaccines [8]. The current COVID-19 pandemic has sped up progress in the field. Two SARS-CoV-2 mRNA vaccines have received accelerated approval by US and EU regulators for clinical use, and several others are undergoing clinical trials [9].

Despite obvious advances in this area over the past few years, many challenges remain for RNA-based therapy and prophylaxis. For siRNAs and miRNAs, eventual off-target effects with silencing of unexpected genes are a concern, despite powerful bioinformatic tools for sequence design [10,11]. For both long and short RNAs, there is a risk of activation of innate immunity by the molecules delivered [12,13]. For all types of RNAs, early degradation in the internal environment of the body is a problem. The half-life of naked siRNA when injected intravenously is short, about 15 min [14]. The stability of naked exogenous mRNA in the body is also low, with half-lives varying between 2 and 25 min [15]. Values can differ to some extent depending on RNA secondary structure [16], the presence of 5’ capping, or the length of the 3’ poly-A tail [17].

Although early work was published demonstrating in vivo protein expression from naked, exogenously delivered mRNA template [18], it is now generally accepted that efficient protein translation from an exogenous mRNA necessarily requires a precursory RNA complexed with a delivery carrier [5].

Currently, there are a number of both viral and non-viral experimental solutions for RNA delivery to cells. Non-viral systems include various cationic polyplexes, lipid nanoparticles, and cell-penetrating peptides (reviewed in [1,19,20]).

Effective RNA delivery vehicles should meet multiple requirements. They should: bind RNA with high capacity and protect it from nuclease cleavage; be reasonably stable, biodegradable, and non-toxic for the organism; and should effectively pass through numerous biological barriers in the body (the last of which usually being a target cell’s endosomal membrane).

In recent years, exosomes have attracted the serious attention of researchers as a very promising nanocarrier for the delivery of short and long RNAs. In terms of cell biology, exosomes are extracellular vesicles secreted by cells, 30–120 nm in size, which mediate intercellular communication. When formed in the endosomal compartment, exosomes selectively include a multitude of biologically active host cell molecules (proteins, lipids, miRNAs, various long RNAs) [21,22]. Subsequently, exosomes transport these molecules to other cells, thereby modulating functional states of recipient cells. Exosomes are found in a range of biological fluids, including blood [23], saliva [24], bronchoalveolar fluid [25], breast milk [26], etc. They are relatively stable, with rather low immunogenicity [27], although the molecules they carry (cytokines, chemokines, miRNAs) are capable of having an immunomodulatory effects [28]. The exosomal membrane effectively protects the cargo from nucleases and proteases of the extracellular environment [29], while surface proteins promote internalization of the exosome into a recipient cell [30]. These characteristics of exosomes make them an inviting candidate vehicle for delivery of a number of drugs, including such difficult and unstable molecules as RNAs.

In this review, we analyze experimental data on the use of exosomes as vehicles for the delivery of therapeutic and prophylactic RNAs. We briefly consider the biogenesis and functions of exosomes, focusing on those biological properties that make them formidable candidates in the race to develop effective delivery carriers. Furthermore, we describe various techniques of cargo loading into exosomes. We then discuss recent publications on the application of exosomes as an experimental means of delivering siRNAs, miRNAs, and long RNAs. Prospects and current challenges in this area of medical biotechnology are covered.

## 2. Biogenesis, Release and Uptake of Exosomes

Unlike other types of extracellular vesicles (microvesicles, apoptotic bodies) which bud directly from the plasma membrane [31], exosomes are formed in the cytoplasm, within structures of the endosomal compartment. Cellular mechanisms of exosome biogenesis provide highly effective selective inclusion of specific molecules of various types (proteins, RNA, lipids).

The precursors of exosomes are formed as secondary invaginations of the endosomal membrane, with their subsequent detachment into the lumen of the endosome and the appearance of a multivesicular body [MVB], containing multiple intralumenal vesicles (ILV) [32]. The molecular mechanisms of exosome biogenesis, and selective sorting of various molecules into them, are being actively investigated, although they are not yet completely understood [33,34]. Currently, two pathways of exosome formation are described, namely, ESCRT-dependent active mechanism and passive, ceramide and CD63-based pathway.

Endosomal sorting complexes required for transport (ESCRT) executes multiple events of cell membrane remodeling, resulting in reverse topology membrane scission [35,36]. ESCRT dependent sorting employs four multiprotein complexes: ESCRT-0, ESCRT-I, ESCRT-II, and ESCRT-III, and several associated accessory components, such as VPS4, VTA1, and ALIX [37]. The key process for protein sorting to the multivesicular body is ubiquitination [38]. Hepatocyte growth factor-regulated tyrosine kinase substrate (HRS) is the ESCRT-0 component which binds the monoubiquitinated proteins and along with clathrin, provides their clustering in specific patches on the endosomal membrane [39]. Subsequently, those ubiquitinated proteins are passed to the ESCRT-I complex. Tumor susceptibility gene 101 (TSG101) is a component of ESCRT-I, which interacts with ubiquitinated proteins and participates in the ESCRT-II activation, causing the formation of secondary invaginations in late endosomes [40]. ESCRT-II supports the movement of ubiquitin-labeled proteins into intraluminal vesicles, acting in cooperation with ubiquitin-removing enzymes. In addition, ESCRT-II recruits ESCRT-III, which participates in the final stage of multivesicular body formation, namely, budding off of intraluminal vesicles [41]. After the formation of MVBs, the ESCRT complex dissociates from them. ESCRT recycling is carried out by the interaction with the AAA-ATPase VSP4 [42].

An ESCRT-independent pathway of exosome formation employs sphingolipids [ceramide], tetraspanin CD63, and heat shock proteins [43,44].

Like protein loading, RNA loading into ILVs also involves several mechanisms. Non-specific, random loading of cellular RNA can occur through RNA binding to the outer (cytoplasmic) surface of the MVB limiting membrane. Incorporation of these RNAs into ILVs is based on the affinity of RNAs to the raft-like regions in the outer layer of the MVB membrane. [45,46]. For non-specific packaging, high abundance of a particular RNA, cytoplasmic localization specifics, and small RNA sizes can increase uptake. However, it is well-known that exosomal RNA profiles significantly differ from those of parent cell [47,48]; this strongly implies the existence of more selective RNA loading pathways.

Currently, molecular mechanisms of exosomal RNA sorting are not completely clear. In general, many aspects of RNA biology including RNA distribution and trafficking depend on interactions with multitude of RNA-binding proteins (RBP) [49,50]. Various RBPs constitute up to 25% of exosomal protein content [51]. RNA-RBP interactions are determined by RNA sequence motifs and/or specific secondary structure. Searches for specific motifs in exosomal mRNAs have revealed several sequences with a high frequency of occurrence [52]. Presumably, these sequences may be related to the selective loading of mRNA into exosomes. Among exosome-enriched miRNAs, a few short motifs apparently involved in targeting (EXO motifs) have also been established by extensive bioinformatic analysis [53]. Obviously, apart from fundamental knowledge, these identified exosome-guiding RNA motifs could be used for the engineering of therapeutic RNAs with exosomal localization [54].

The involvement of several RBPs in the loading of RNA into exosomes has been experimentally demonstrated, including Annexin A2, heterogeneous nuclear ribonucleoproteins A2/B1 (HNRNPA2B1) [55], YBX1 [56], ALIX [57], major vault protein (MVP) [58], and SYNCRIP [59]. Some other factors, such as RNA/RBP modifications (sumoylation, phosphorylation, uridylation, ubiquitylation) [55,60], have also been demonstrated to be of significance in a complex process of RNA guidance to forming exosomes.

Upon completion of MVB formation, it either fuses with a lysosome [61], or is transported to the cell surface and fuses with the plasma membrane, with ILV release as exosomes [32]. The molecular mechanisms that determine the MVB’s fate are rather unclear. There is definitely a tight interplay between lysosome function and exosome production. Apparently, there are lysosomes which regulate the balance between MVB degradation and exosome release [62]. Release of exosomes is regulated by several small GTPases, such as Rab7 [63], Rab11 [64], Rab35 [65], and Ral [66,67]. Membrane fusion is mediated by SNARE proteins, such as VAMP7 [68] and YKT7 [69].

Knowledge of the mechanisms of recipient cell exosomal uptake is of great importance for the development of exosome-based delivery vehicles. Clearly, natural exosomes are capable of preferentially binding specific target cell types and inducing specific phenotypic or biochemical changes. For instance, exosomes from primary neurons are internalized only by other neurons, whereas those from neuroblastoma cells are internalized predominantly by glial cells [70]. Similarly, exosomes derived from various cell types (mesenchymal stromal cells (MSC), monocytes), when administered to a co-culture of both cell types, are captured only by homotypic cells [71]. This specificity is based on the protein repertoire exposed on the surfaces of both the exosome and recipient cell. These molecules include, for instance, integrins [72]; heparan sulfate proteoglycans [73]; T cell immunoglobulin and mucin domain containing protein 4 (Tim4) [74]; and sialoadhesin CD169 [75].

Exosomal internalization by recipient cells apparently occurs via several endocytic pathways, such as a clathrin-dependent pathway; a caveolin-dependent pathway; macropinocytosis; and a lipid raft-mediated pathway. Direct membrane fusion is also possible (reviewed in: [30]). While direct fusion provides immediate cytosolic delivery of exosomal cargo, endocytosed exosomes enter the recipient cell’s endosomal compartment. It is assumed that their escape from the endosome lumen by membrane fusion is promoted by low endosomal pH [76].

**Figure 1:**
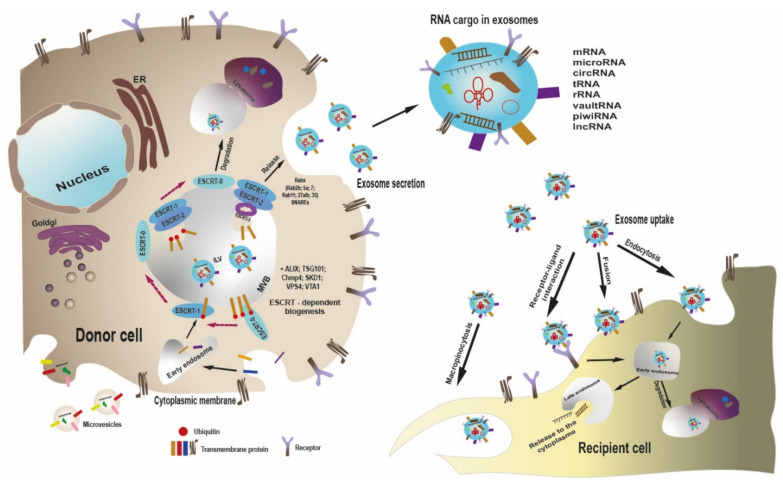
Scheme of exosome biogenesis, release and uptake by recipient cell. Unlike microvesicles, which bud directly from the plasma membrane, exosomes are formed within the late endosome. When forming exosomes, ESCRT-0 binds the monoubiquitinated proteins, clusters them in specific patches on the endosomal membrane and passes to ESCRT-1. ESCRT-1 interacts with ubiquitinated proteins and activates ESCRT-2, which leads to formation of secondary invaginations of the late endosome and movement of ubiquitin-labeled proteins into ILV. ESCRT-2 recruits ESCRT-3, which exerts budding off of ILV, with MVB formation. MVB is subjected to either lysosomal degradation or plasma membrane fusion, with release of ILV as exosomes. A number of Rab GTPases and SNARE proteins are involved into exosome secretion. Exosomes contain a specific set of proteins and RNAs of several types. Exosomes can interact with recipient cell in several ways, such as receptor–ligand interaction, direct membrane fusion, macropinocytosis, or endocytosis. Within recipient cell, exosomes are either degraded or release their cargo to the cytosol, thereby modifying the recipient cell functions.

The essential steps of biogenesis, release and uptake of exosomes are summarized in Figure 1.

## 3. Why Exosomes Are Advantageous for Therapeutic RNA Delivery

Exosomes are natural containers which perform cell-to-cell transport of multiple biologically active molecules including several types of RNA. Progress in exosome purification methods (reviewed in: [77,78]), as well as technical developments in transcriptomics, have made detailed analysis of exosomal nucleic acids possible. It was found that exosomes carry many RNA species of various lengths, such as mRNAs [79,80], miRNAs [29,81], lncRNAs [82], circRNAs [83,84], small nuclear RNAs (snRNAs), small nucleolar RNAs (snoRNAs), tRNAs, rRNAs and some other less represented RNA types [85,86,87].

It has been firmly established by extensive research [88,89] that a significant fraction of these RNA molecules: (i) is specifically loaded into donor cell exosomes; (ii) is passed to recipient cells intact; and (iii) when passed, modulates recipient cell function. Taken together, these characteristics make exosomes a nearly ideal biomimetic carrier for RNA-based therapeutics. The ability of exosomes to transfer and specifically deliver various RNA types to target cells [90] is of great importance in several areas. These include understanding of the fundamental mechanisms of multiple diseases and developing approaches for their treatment and prevention, including gene therapy.

Crucially, exosomes overcome the immunogenicity issue, which is an insurmountable hurdle for many other delivery vehicles [27,91]. Exosomes exhibit longer sustained circulation in mice, compared to liposomes, due to the surface presence of CD47, a molecule that prevents fast capture by monocytes or tissue macrophages [92]. The small size of exosomes (30–120 nm) also diminishes their clearance rate by macrophages. In addition, exosomes are small enough to pass through fenestrae in liver capillaries [93], which permits extravasation. Obviously, effective extravasation is a key step for exosome-delivered cargo to exert its effects.

Exosomal surfaces can be engineered to enhance their targeting to certain cell types. For example, exosomal membrane protein Lamp2b has been fused to the rabies virus RVG peptide which is known to bind specifically to neurons. In result, RVG-exosomes acquired the ability to cross the blood–brain barrier in mice and deliver siRNA therapeutics to brain tissue [94]. Similarly, exosomes have been engineered to carry Epstein–Barr virus glycoprotein 350. These exosomes specifically bind CD19+ B cells, but not other peripheral blood mononuclear cells [95].

In another work, HEK293 cells were modified to stably overexpress the GE11 peptide fused with the transmembrane domain of the platelet-derived growth factor receptor. GE11 binds specifically to the epidermal growth factor receptor (EGFR), which is abundantly expressed in tumors of epithelial origin. GE11-containing exosomes from these donor cells were able to deliver therapeutically relevant let-7a miRNA specifically to EGFR-expressing xenograft breast cancer tissue in RAG2^−/−^ mice [96].

Although the aforementioned recombinant protein-based technology for exosome targeting is effective, it is also very expensive, time consuming and labor intensive. Therefore, simpler, click chemistry approaches to exosomal surface modification have been developed. In this way, functional groups are attached to the exosomal surface via covalent bonds. Typically, exosome modification by click chemistry is based on a reaction between alkyne and azide groups (copper-catalyzed azide alkyne cycloaddition), which forms a triazole linkage [97]. By click chemistry, exosomes were functionalized with RGE peptide targeting neuropilin-1. As a result, these exosomes, loaded with curcumin, displayed high tropism for glioblastoma cells and featured positive therapeutic effects [98].

Another approach to adding required molecules to exosomal surfaces, called the ‘post-insertion’ technique, entails exosome mixing with lipid micelles conjugated to the molecules of interest. For example, anti-EGFR nanobodies have been fused to PEGylated phospholipids. Subsequent mixing of these nanobody-PEG-lipid micelles with exosomes at elevated temperatures resulted in effective transfer of nanobody-PEG-lipids to the exosomal membrane [99]. In general, this “exosome mimetic” strategy is capable of providing a wide range of exosomal surface modifications [100].

Apart from exosomal surface remodeling, cargo RNA can be engineered in various ways to achieve efficient packaging into exosomes. For example, incorporation of a “zipcode” (homing) sequence into the 3′ UTR of the cargo RNA enables recognition by specific RBPs associated with MVB-targeting [101].

Despite all advantages of exosomes listed above, their clinical implementation for the drug delivery (including RNA delivery) is still challenging. First, safety concerns should be addressed more rigorously, including potential immunogenicity and tumorigenicity of exosomes. Naturally, exosomes are biologically active entities aimed at the intercellular signaling, particularly within the immune system. Although shown to be less immunogenic than many artificial carriers, exosomes inherently exert immunomodulatory effects, depending on the donor cell type. Therefore, the appropriate choice of the producing cells is of key significance for the exosome application. Most often, autologous dendritic cells (DC), macrophages, mesenchymal stromal cells (MSC), fibroblasts, as well as HEK293-T cells are utilized as exosome donors [102,103]. Of note, MSC-derived exosomes are capable to promote the tumor vascularization [104], while mature DC-derived exosomes have immunostimulatory properties [105].

The second obstacle currently complicating the clinical implementation of exosomes is the drawback of purification technologies, which leads to instability in the composition of exosome preparations. In terms of the physico-chemical properties (size, buoyant density, molecular composition), exosomes partially overlap with other nanocomponents of the cellular secretome, which fundamentally hinders their reproducible separation in a completely pure form. There is a significant discrepancy in the assessment of the composition of exosomal proteins and RNAs, depending on the purification methods used by the researchers [22]. Obviously, these differences in molecular composition further affect the functional impact of the exosome preparation on recipient cells during exosomal delivery of therapeutic RNAs.

Finally, the problem of scaling up the production of exosomes for clinical purposes has not yet been solved. The level of exosome secretion by producer cells of any type is low, and scaling from research to clinical practice incurs huge material and labor costs. In this sense, ultracentrifugation and polymer precipitation are the simplest and most productive methods for isolating exosomes, but they rather do not provide a sufficient purity of the preparation. More complex approaches, such as gradient fractionation, size exclusion chromatography, and especially antibody-based affinity separation, make it possible to obtain more purified products, but their cost is much higher, and the yield of exosomes is lower, which makes technological scale-up difficult. The search for an acceptable compromise is ongoing, and, clearly, the further research in the field is required.

To summarize, exosomes are biocompatible, non-toxic RNA delivery vehicles with low immunogenicity. They are able to provide: extravasation of cargo RNA; internalization by target cells; and endosomal escape. There are broad opportunities for the functionalization of exosomal surfaces to increase delivery targeting, and various methods for modification of RNA cargo also exist. These features make exosomes a highly promising carrier for the delivery of both therapeutic RNAs [106] and prophylactic RNA vaccines [107,108], although extensive further research is necessary on the donor cell selection, exosome safety, optimization and scaling-up of purification protocols, before exosome-based drug delivery is implemented into clinics.

## 4. Techniques for RNA Loading into Exosomes

Methods for RNA loading into exosomes include both: straightforward approaches for RNA incorporation into previously isolated, purified exosomes; and more sophisticated cellular engineering techniques. In the latter, expression of the target RNA by exosome-producing cells, and the intracellular transport of this RNA into the forming exosomes, occur first. Isolation and purification of target RNA-containing exosomes then proceeds.

In principle, the simplest method of loading into the lumen of an exosome is a mere co-incubation [exosomes with cargo molecules], during which molecules diffuse through the lipid bilayer of the exosome. This approach permits sufficiently effective penetration into exosomes for some therapeutically important hydrophobic substances, including doxorubicin [109], curcumin [110], and paclitaxel [111]. In contrast, however, RNA of any type is a negatively charged molecule, which precludes its spontaneous diffusion through the exosomal membrane’s lipid bilayer. In order to improve RNA’s interaction with the exosomal membrane, chemical modifications can be performed. Promising results have been obtained by conjugation of siRNA with cholesterol [112,113]. This makes RNA molecules more lipophilic and ensures their efficient incorporation into exosomes.

Alternatively, pore formation in the lipid bilayer for the entry of RNA into the exosome is possible. Several physical methods use such a mechanism. The most commonly used option for membrane pore formation is exosome electroporation, in which a mixture of exosomes and cargo RNA is pulsed with an electric field. Electroporation parameters require optimization depending on donor cell type. Typically, applied voltages range from 150 to 700 V [114,115], with exosome concentrations ranging from 0.07 to 0.5 μg/μL [5,94,116]. There are a number of reports on the successful loading of siRNAs [94,117,118,119,120,121] and miRNAs [122,123,124,125] into exosomes by electroporation, followed by in vitro or in vivo small RNA delivery into cells, with silencing of target genes. Cumulative experience, however, has also revealed some disadvantages of exosome electroporation, such as damage to vesicle integrity, aggregation, and low RNA loading efficiency [126]. Some authors did not achieved satisfactory loading of small RNAs by exosome electroporation [127]. Most fluorimetry-based estimates of small RNA loading efficiency into electroporated exosomes indicate about 20–25% of small RNA is captured by exosomes [116]. Furthermore, according to Kooijmans et al. [126], electroporation leads to massive aggregation of siRNA-containing exosomes (obviously not suitable for subsequent siRNA delivery). Trehalose medium minimizes the aggregation [128]. When using it, the value of RNA capture decreases to 0.05% [126]. This low loading efficiency significantly increases material consumption and the final cost of preparing of RNA-containing exosomes. Data on the applicability of electroporation for loading longer mRNAs into exosomes are also inconsistent. In one publication, Cas9 mRNA (4521 nucleotides) was successfully loaded into exosomes using electroporation with an insertion efficiency of 18% [129]. Other authors, however, have reported the impossibility of HChrR6 mRNA inclusion into exosomes by electroporation and the need to use cell-based technologies for loading for this purpose [130].

In addition to electroporation, other simple methods have been used for the formation of pores in the exosomal membrane to load small RNAs, such as sonication [131] and freeze-thaw cycles [132].

Further options are provided by: repeated extrusion of mixtures (exosomes, cargo molecules) through a syringe-based lipid extruder with 200 nm or 400 nm pore size [133,134]; or by exosome treatment with the pore-forming agent saponin [135,136]. Currently, these methods are used for loading of rather small cyclic compounds than for RNA.

Another approach that has been successfully applied for loading RNA into exosomes is transfection of exosomes using commercial liposomal reagents (HiPerFect, Lipofectamine, ExoFect), with formation of exosome/liposome hybrids. Exosome/liposome fusion occurs via co-incubation and can be further enhanced by repeated freezing-thawing of the mixture [137]. The exosome/liposome hybrid approach has been applied to exosome loading with miRNAs [138,139,140] and siRNAs, [141,142]. Importantly, according to published data, transfection of exosomes with lipid carriers is feasible not only for the inclusion of short RNAs, but also for much longer molecules, such as: mCherry mRNA (approx. 800 nucleotides) [143]; Antares2 mRNA (2000 nucleotides) [144]; as well as massive CRISPR–Cas9 plasmid expression vectors [145].

However, the exosome/liposome hybrid approach has also drawn criticism. After co-incubation, separation of exosomes from unbound lipid vesicles is either not carried out at all [139,140], or is performed by filtration. Such filtration is based on assumed differences in the linear sizes of exosomes and liposomes [141]. However, the two types of vesicles not only coexist in solution, but actively interact leading to hybrid formation. Therefore, taking also into account the generally high lability of lipid systems, complete separation is fundamentally impossible. Transfection reagents themselves are capable of binding RNA and delivering it to cells. Therefore, interpreting the results of RNA (or DNA) delivery into cells by exosome/liposome hybrids requires caution, especially as it relates to long mRNA molecules and CRISPR-Cas9 expression plasmids. The exosome is not a hollow vesicle, but is filled with a range of endogenous proteins and RNAs; this complicates incorporation of exogenous plasmids or mRNA into it.

The aforementioned physical and chemical methods deal with isolated exosomes. Cell-engineering methods involve delivery of the target RNA into donor cells, followed by incorporation of this RNA into exosomes during their biogenesis. Several cell-engineering approaches of this kind have been proposed.

1. In the simplest case, cells are transfected directly with target RNAs: miRNA, [146,147]; siRNA [148]; antisense miRNA antagonist [149]; or in vitro transcribed mRNA [150]. Then, the loading of transfected RNAs into exosomes occurs. After harvesting and purification of exosomes secreted by donor cells, exosome-based RNA delivery into target cells has been demonstrated, including therapeutically relevant functional effects of RNAs on the recipient cells.
2. Transfection of plasmids, or viral transduction, can be performed to express target RNAs in donor cells, followed by incorporation of these RNAs into exosomes. This approach can be implemented for both miRNA [151,152,153] and long mRNA [154,155,156,157].

In the two cases above, the RNA (delivered directly to cells, or expressed from a plasmid or viral vector) does not carry any exosome-guiding sequence. Apparently, its loading into the exosomes of the donor cell from the cytoplasm occurs through non-specific engulfment as part of secondary invaginations of the endosomal membrane during the MVB formation. With this in mind, the abundance (number of copies) of RNA cargo available for loading is critical. Expression plasmids and viral vectors typically provide a high level of transcription, while the cytosolic concentration of directly delivered RNAs (both small RNAs and mRNAs) may be insufficient for its efficient packaging into exosomes of a donor cell. In addition, exogenous RNA delivered to the cytoplasm arrives at the general pool of cellular RNAs and inevitably enters cellular functional RNA pathways, such as gene silencing (for small RNAs), protein translation (for mRNAs), and, finally, degradation pathways. This can affect the state of donor cells, including the production/composition of their exosomes. As known, degradation of exogenous RNA can be slowed down by introducing chemically modified nucleotides into it [158].

3. To overcome the aforementioned limitations, more specifically, RNA sequence and a protein that binds and directs it to exosomes can be engineered. For example, a C/D box can be inserted into the 3’ UTR of the target mRNA, which is recognized by archaeal ribosomal protein L7Ae. In turn, the L7Ae sequence is fused with the C-terminus of the CD63 exosomal marker protein. Co-transfection of these two constructs provides highly specific targeting of the mRNA of interest to exosomes [159].

A similar principle was implemented in the Targeted and Modular EV Loading (TAMEL) platform proposed by Hung and Leonard [101]. In it, exosomal proteins (Lamp2b, or CD63) were fused with MS2 bacteriophage coat protein capable of binding a certain type of RNA secondary structure (MS2 stem loop). Overexpression of these fusion proteins in cells, and simultaneous lentiviral transduction of model dTomato mRNA (1.8 kb, containing several MS2 stem loops), resulted in efficient, targeted delivery of this mRNA to exosomes.

**Figure 2:**
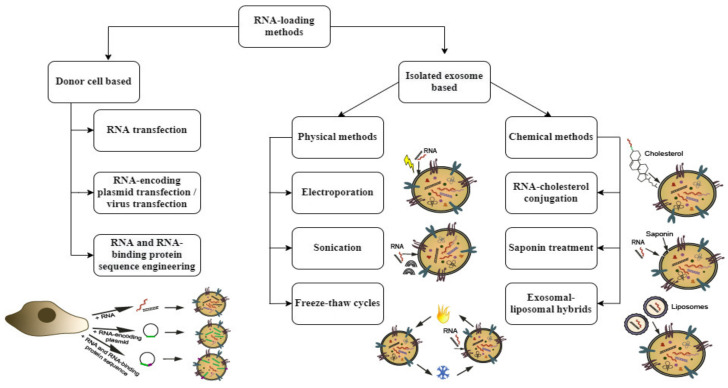
Techniques for RNA loading into exosomes. RNA loading into exosomes can be achieved by either exosome donor cell-based or isolated exosome-based approaches. Donor cell-based methods involve target sequence delivery to the exosome donor cell via direct RNA delivery or via plasmid transfection or viral transduction, with the following intracellular packaging of target RNA to exosomes and further exosome harvesting. Loading into exosomes can be enhanced by RNA sequence engineering, including zipcode sequence or specific secondary structure joining. RNA-binding proteins (RBPs) can be engineered too, to recognize these RNA motifs. Isolated exosome-based methods are the several physical methods for exosomal membrane poration (electroporation, sonication, freeze-thaw cycles), as well as chemical methods for RNA penetration through the exosomal membrane. Chemical methods include strengthening of RNA affinity to the exosomal membrane via RNA-cholesterol conjugation, saponin-based pore formation and liposome-mediated RNA packaging into exosomes (exosome-liposome hybrid method).

Various techniques for RNA loading into exosomes are summarized in Figure 2.

## 5. Exosomal Delivery of Therapeutically Relevant RNAs

The first results on successful exosome-mediated siRNA delivery to target cells and subsequent specific gene silencing in them were obtained a decade ago [94,114]. Alvarez-Erviti et al. engineered exosomes to carry Lamp2b-RVG peptide, electroporated BACE1 siRNA into them and achieved a significant decrease in BACE1 expression in mouse brain tissue by systemic injection of these exosomes [94]. Walgreen et al. performed siRNA electroporation into plasma exosomes from the peripheral blood of healthy donors and demonstrated selective gene silencing of MAPK-1 in the target human monocytes and lymphocytes [114].

In recent years, advantages of exosomes as carriers have been repeatedly used experimentally in works on the delivery [both in vitro and in vivo] of therapeutically significant RNAs (most often small RNAs, such as siRNAs, miRNAs, miRNA antagonists) aimed at treating a number of diseases, such as oncology, neurological and neurodegenerative diseases, and infections.

Below, we consider the most substantial experimental results of in vivo delivery of therapeutically important RNAs by exosomes, in order to characterize the current state of the art.

### 5.1. siRNAs

Successful in vivo experiments on the use of exosome-delivered therapeutic siRNAs have in most cases been aimed at various neurological diseases, including Huntington’s disease, Alzheimer’s disease and spinal cord injury, as well as tumors of various origins. In the case of neurological diseases, RVG-Lamp2b modified exosomes were predominantly used to overcome the blood–brain barrier (BBB) and blood–spinal cord barrier (BSCB) and to deliver siRNA to the brain and spinal cord. In oncological diseases, targeting of siRNA-containing exosomes to tumor tissue can be achieved by modifying them with the RGD-LAMP2b fusion protein. RGD peptide has a high affinity to integrin αvβ3 and specifically delivers exosomes to tumors in vivo [160]. For cancer treatment, siRNA targets include genes critically important for growth of tumors, such as S100A4, c-Met, PAK4, KRAS, etc. Of note, currently a phase 1 clinical trial of metastatic pancreatic adenocarcinoma treatment by MSC-derived exosomes with siRNA to mutated KRAS is in progress [NCT03608631].

Recent data on exosomal delivery of therapeutic siRNA in vivo are summarized in Table 1.

### 5.2. miRNAs

Dysregulation of miRNA profiles is a key component of a wide range of diseases including those of a cardiovascular, infectious, oncological, or neurological nature. Compensation for these pathological disturbances is a promising therapeutic strategy. Unlike siRNAs, miRNAs have multiple targets in the genome to perform “fine tuning” of gene expression. Exosomes are natural carriers which transfer a multitude of donor cell miRNAs. These endogenous exosomal miRNAs themselves can be of high therapeutic significance. For example, MSC-derived exosomes contain more than 150 pre-miRNAs and miRNAs [170]. Such exosomes have shown therapeutic potential in the treatment of multiple diseases including liver fibrosis, acute kidney injury, myocardial infarction, Alzheimer’s disease, type 1 diabetes, and asthma. Several clinical trials (phases I, II, III) of MSC exosome–based therapies are now ongoing [171]. For severe COVID-19, MSCc exosome treatment resulted in reliable improvement in patient oxygenation, neutrophil count reduction, and declines in acute phase reactants (C-reactive protein, ferritin, D-dimer) [172,173], and the key role of several miRNAs (miR-126, miR −30b–3p, miR −145, miR −27a–3p) has been established [174].

In addition, exosomes from various cells (most often MSCs) with exogenously loaded miRNAs have been repeatedly tested experimentally to treat cancer and several neurological disorders, with proven amelioration in disease course. Table 2 represents data on the experimental treatment of various types of cancer and neurological disorders by exosome-delivered miRNAs.

### 5.3. mRNAs

Although the huge potential of mRNA-based therapy is widely acknowledged, current experience in delivery of therapeutic mRNAs by exosomes is rather limited. The large size of mRNA significantly hampers its incorporation into exosomes, in comparison with small RNAs. There is one publication that deals with model mRNA encoding fluorescent protein, loaded into exosomes by exosomal-liposomal hybrid method [143].

More therapeutically relevant, sophisticated cellular engineering techniques of specific mRNA design and targeting are typically used. Maugeri and co-authors delivered mRNA encoding human erythropoietin (hEPO-mRNA) to epithelial cells HTB-177 using LNP. Then, hEPO-mRNA was revealed in EVs secreted by these donor HTB-177 cells. After intravenous administration of hEPO-mRNA-containing EVs to mice, hEPO protein was detected in plasma and number of organs in 2 h after injection [180].

In Kojima’s group work, catalase mRNA fused with C/Dbox for effective exosomal targeting was engineered [159]. In this study, designer exosomes carrying RVG-Lamp2b effectively delivered catalase mRNA across the BBB, and finally decreased 6-OHDA-mediated neuroinflammation in mouse model of Parkinson’s disease.

In another study, exosomes aimed at HER2+ve breast cancer treatment were developed. For exosome targeting to the cancer cells, HEK293 cells were transfected with plasmid encoding EVHB chimeric protein, which contains high-affinity anti-HER2 scFv antibody. This protein is capable of being exhibited on the EV surface. These cells were also transfected with a plasmid-encoding modified *E. coli* enzyme HChrR6. This enzyme is capable of converting the prodrug (CNOB) into the cytotoxic drug (MCHB). In transfected cells, HChrR6 mRNA was loaded into HER2-targeting exosomes. When administered in vivo, these exosomes caused effective HChrR6 expression in tumor cells, with subsequent near-complete growth arrest of BT474 xenografts after CNOB prodrug treatment [130].

For Shwannoma treatment, 293-T cells were stably transfected with plasmid encoding a cytosine deaminase (CD) fused with uracil phosphoribosyltransferase (UPRT) These enzymes activate “cell suicide” upon administration of the prodrug 5-fluorocytosine (5-FC), which is converted to 5-fluorouracil (5-FU). Exosomes released by these donor cells contained CD-UPRT mRNA. When injected into mouse shwannoma xenografts, in combination with systemic prodrug delivery, exosomes provided significant regression of these tumors [154].

In a recent publication by Tsai et al., exosome-packaged mRNA encoding the S and N proteins of SARS-CoV2 was used to experimentally vaccinate mice. The results obtained by the authors indicate the post-vaccinal appearance of specific anti-S and anti-N antibodies in the blood plasma, as well as T cell responses to N and S proteins [144]. These data confirm the possibility of using exosomes as a means of delivering COVID-19 mRNA vaccines.

Thus, exosome-mediated delivery of mRNAs is of proven therapeutic relevance. At the same time, however, complexities present with loading and targeting technologies based on cell engineering complicate clinical implementation. Table 3 summarizes data on the exosome-based mRNA delivery.

## 6. Conclusions and Future Perspectives

Exosomes are natural nanocontainers actively secreted by the body’s cells, thereby transmitting molecular signals of various types to recipient cells. Cellular mechanisms of exosome biogenesis involve specific sorting of RNA and proteins for incorporation. As a result, the molecular composition of exosomes is closely related to the functional state of the donor cell; this makes exosomes important diagnostic and prognostic markers in a number of diseases (primarily oncological). The ability of exosomes to protect their cargo from degradation in the extracellular environment, and to transfer it to recipient cells, makes them effective candidates for the delivery of RNA in therapeutic or prophylactic regimes. The possibility of exosomal surface functionalization enables improved delivery targeting.

There is no doubt about the significant potential of exosomes as a carriers of gene therapy agents. In recent years, there has been an explosive growth in research interest in exosome-based drug delivery, and several clinical trials of exosome-encapsulated siRNAs are in progress currently. However, there are certain obstacles to the more active implementation of exosome technology in clinical practice.

Despite their proven low immunogenicity, exosomes are always biologically active entities aimed at modulating recipient cell function. For example, tumor-derived exosomes prepare a niche for metastasis [181]. In this context, safety concerns around exosome-based therapies need to be addressed more thoroughly.

The question of choosing the type of donor cells requires special attention. Exosomes are not biologically inert particles, but perform regulatory and communicative (including immunomodulatory) functions, and their activity depends on the type and functional state of parental cells. Currently, human MSC, immature DC, and HEK293-T cell lines are most often used as exosome donors. In the future, a “universal exosome donor” could be developed, which is optimal for packaging and delivery of various RNA cargoes. In this sense, of great interest is the work of Kojima and colleagues [159], who created an “exosome production booster” based on HEK293-T, suitable for further engineering of exosome-targeted RNAs of interest.

Clearly, further development of specific ligands that determine the dispatching of exosomes to tissues and target cells is necessary. In this regard, there are examples of striking success, such as RVG peptide-based targeting of exosomes to neuronal tissue [94] or GE11peptide-based targeting of exosomes to EGFR-positive tumors [96]. However, a huge therapeutic potential of exosome-encapsulated RNAs definitely requires a significant expansion of this list. Apparently, monoclonal antibody-based aiming of exosomes could be a promising, albeit expensive, solution.

The development of a technology for loading of RNAs into exosomes cannot be considered resolved. Plain pore-forming approaches dealing with isolated exosomes are attractive due to their methodological simplicity; however, their usage has often resulted in uncontrolled aggregation and damage to the integrity of both the exosomes themselves and their cargo, especially long mRNA molecules. In general, cell engineering methods seem to be more promising, although currently expensive and difficult to scale.

Exosome purification technology and its scalability is also challenging at the moment. It is well known that the vesicular secretome of the cell is heterogeneous. EVs of different types differ significantly in biogenesis, molecular composition, and functions. It is probable that some of them are “debris bags” for the cell, as was postulated in early studies, while others perform specific communicative tasks. At the same time, vesicles of different types overlap in size and buoyant density, and reproducibly obtaining completely purified homogeneous preparations of exosomes is currently extremely complicated. The most effective and specific purification method is the immunoaffinity technique; however, the high cost of antibodies makes it rather difficult to scale it for clinical application. Undoubtedly, further efforts of researchers are needed to optimize exosome purification technologies and their industrial scaling.

Finally, more fundamental knowledge is needed regarding the biological roles of exosomes in the body including: kinetics; overcoming of (or limitation by) biological barriers; and molecular mechanisms of endosomal escape. The roles of multiple molecules delivered by exosomes have not been sufficiently studied. For example, the functions of a number of exosomal lncRNAs and circRNAs remain rather elusive, yet such understanding is obviously necessary to realize the therapeutic potential that exosomes represent.

## Figures and Tables

**Table 1:** Exosome-mediated in vivo delivery of therapeutically relevant siRNAs.

Donor Cell Type	siRNA Cargo	Exosome Loading Procedure	Administration Route	Target Organ	Effect	Ref.
Cancer						
HEK293-T cells overexpressing an iRGD peptide fused with Lamp2b	siRNA to KRAS	cell transfection with siRNA	i/v	A549 cell-based mouse tumor xenograft.	specific targeting of iRGD-exosomes to tumor tissues, strong inhibition of tumor growth	[160]
Autologous breast cancer cells	siRNA to S100A4	CBSA/siRNA nanoparticles were first prepared by incubation. Then, CBSA/siRNA were coated by exosomal membrane, by incubation and extrusion method	intravenously (i/v)	mouse lung metastasis	decrease in metastatic nodules. S100A4 (metastasis-related protein) silencing, by Western blot analysis and fluorescence microscopy.	[161]
HEK293-T cells	siRNA to c-Met	cell transfection with siRNA	i/v	gastric tumor mouse xenograft	decrease in expression of c-Met (a key driver for carcinogenesis)	[162]
PANC-1 cells	siRNA to PAK4	electroporation	intra-tumoral injection	PANC-1 mouse tumor xenograft	reduced tumor growth and enhanced mice survival, decrease in PAK4 (a driver of pancreatic cancer progression) by IHC staining of tumors.	[120]
HEK293-T cells overexpressing a human IL3 fragment fused with Lamp2b	siRNA to BCR-ABL	cell transfection with siRNA	intraperitoneal injection	CML mouse xenograft	slower tumor growth, reduction in BCR-ABL mRNA	[163]
MSCs	siRNA to GRP78	cell transfection with siRNA	injection around the tumor, introperitoneal injection	HCC mouse xenograft and metastasis	inhibition of the growth and metastasis of the cancer cells.	[164]
Neurological disorders						
Dendritic cells overexpressing RVG peptide fused to Lamp2b	siRNA to BACE1	electroporation	i/v	mouse brain	62% knockdown of BACE1, a therapeutic target in Alzheimer’s disease	[94]
U87 cells	cholesterol-conjugated siRNA to Huntingtin mRNA	incubation at 37 °C	unilateral infusion into striatum	mouse striatum	silencing of up to 35% of Huntingtin mRNA, a target of Huntington disease	[165]
MSC	siRNA to CTGF	electroporation	i/v	rat injured spinal cord (ISC)	significant decrease in CTGF expression in ISC segment, with reductions in inflammation and neuronal apoptosis	[166]
HEK293-T cells overexpressing RVG peptide fused to Lamp2b	siRNA to HMGB1	electroporation	i/v	ischemic stroke model by middle cerebral artery occlusion (MCAO)	decreases in: HMGB1; tumor necrosis factor-α (TNF-α); apoptosis; and size of affected brain region	[119]
Primary rat corticalneuronal cultures	siRNA to ASC	electroporation	femoral artery injection	rat ISC	knockdown of ASC protein levels by approx. 76%, decrease in caspase-1 activation and processing of IL-1β after SCI.	[167]
Dendritic cells overexpressing RVG peptide fused to Lamp2b	siRNA to Alpha-synuclein	electroporation	i/v	mouse brain	decrease in alpha-synuclein expression (a target of Parkinson disease) in normal and S129D a-Syn transgenic mice	[168]
HEK293-T cells overexpressing RVG peptide fused to Lamp2b	siRNA to MOR opioid receptor	cell transfection with siRNA	i/v	mouse brain	reducing in MOR mRNA and protein, with inhibition of morphinerelapse	[169]
Infection						
Huh7 cells	siRNA to CD81	transfection of shRNA-encoding plasmid	i/v injection of 100-fold concentrated shCD81Huh7 -conditioned medium	mouse liver	20% reduction in cell surface expression of CD81, a HCV receptor	[153]

**Table 2:** Exosome-mediated in vivo delivery of therapeutically relevant miRNAs.

Donor Cell Type	miRNA Cargo	Exosome Loading Procedure	Administration Route	Target Organ	Effect	Ref.
Cancer						
HEK293 cells overexpressing GE11 peptide fused with the transmembrane domain of platelet-derived growth factor receptor	Let-7a	cell transfection with miRNA	i/v	HCC70 cells mouse breast cancer xenograft	tumor growth suppression	[96]
MSC	miR-122	cell transfection with miRNA-encoding plasmid	intra-tumor injection	HepG2 cells mouse xenograft	downregulation of CCNG1, IGF1R, and ADAM10, upregulation of Caspase 3 and Bax genes in tumors. When administered with sorafenib, tumor growth suppression.	[175]
MSC	miR-146	cell transfection with miRNA-encoding plasmid	intra-tumor injection	9L glioma cells rat xenograft	tumor growth suppression	[151]
hepatic stellate cell LX2	miR-335-5p	lipofectamine-based exosome transfection	intra-tumor injection	MHCC97H hepatocellular carcinoma cells mouse xenograft	tumor shrinkage	[138]
MSC	miR-124	cell transfection with miRNA-encoding lentiviral vector	intraperitoneal, intra-arterial, intratumoral	GSC267 glioma cells mouse xenograft	50% of animals living long term, complete regression of tumors, FOXA2 inhibition	[176]
Neurological diseases						
MSC	miRNA-29b	cell transfection with miRNA-encoding lentiviral vector	i/v	rat ISC	SCI improvement, increased hind limb motor function, rise in NF200 and GAP-43 positive neurons number, decreased contractile nerve cell numbers and GFAPpositive neurons	[177]
MSC overexpressing RVG peptide fused with Lamp2b	miR-124	electroporation	i/v	mouse local cortex ischemia	decreased Gli3 and Stat3 mRNA, decreased Sox2 and Nestin expression; promotion of neurogenesis	[178]
MSC	miR-124-3p	cell transfection with miRNA-encoding plasmid	i/v	M2 macrophages in spinal cord ischemia-reperfusion injury (SCIRI)	decreased Ern1 expression, enhanced M2 macrophage polarization, inhibition in SCIRI-induced cell apoptosis, SCIRI amelioration	[179]

**Table 3:** Exosome-mediated delivery of therapeutically relevant mRNAs.

Donor Cell Type	mRNA Cargo	Exosome Loading Procedure	Administration Route	Target Organ	Effect	Ref.
HTB-177 cells	human erythropoietin (hEPO) mRNA	transfection of donor cells with LNP transferring mRNA	i/v	mouse heart, lung, spleen, liver, kidney, thymus, pancreas, brain	hEPO protein production was detected in plasma and organs of injected mice in 2 h after injection.	[180]
HEK-293T cells overexpressing exosome production booster and RVG peptide fused to Lamp2b	catalase mRNA fused to C/Dbox	plasmid transfection of donor cells	subcutaneous implantation of exosome donor cells with Matrigel	mouse brain	decreased 6-OHDA-mediated neuroinflammation in Parkinson’s disease model	[159]
HEK-293 cells overexpressing EVHB- anti-HER2 scFv antibody chimeric protein.	mRNA of HChrR6 with EV-loading zipcode sequence	plasmid transfection of donor cells	intraperitoneal injection	mouse BT474 breast cancer xenograft	HChrR6 expression in tumor cells, with subsequent near-complete growth arrest of BT474 xenografts after prodrug CNOB treatment	[130]
HEK-293T cells	mRNA of cytosine deaminase (CD) fused with uracil phosphoribosyltransferase (UPRT)	plasmid transfection of donor cells	intratumoral injection	mouse shwannoma xenograft	in combination with systemic delivery of prodrug 5-fluorocytosine, significant inhibition of schwannoma growth	[154]
293F cells	SW1 mRNA encoding Spike protein, LSNME mRNA encoding Nucleocapsid protein and fragments of thespike, membrane, and envelope proteins of SARS-CoV-2	cationic lipid bound mRNA was transfected to exosomes	intramuscular injection	mouse blood plasma	vaccination-induced specific anti-N and anti-S antibody responses, and T cell responses to N and S proteins.	[144]

## Abbreviations

MVB—multivesicular body, ESCRT—endosomal sorting complexes required for transport, RBP—RNA-binding protein, MSC—mesenchymal stromal cell, lncRNA—long non-coding RNA, circRNA—circular RNA, snRNAs—small nuclear RNA, snoRNAs—small nucleolar RNA, RVG—rabies virus glycoprotein, EGFR—epidermal growth factor receptor, PEG—polyethylene glycol, DC—dendritic cell, EV—extracellular vesicle, TAMEL—targeted and modular EV loading, BBB—blood–brain barrier, BSCB—blood-spinal cord barrier, CBSA—cationic bovine serum albumin, IHC—immunohistochemistry, CML—chronic myeloid leukemia, HCC—hepatocellular carcinoma, ISC—injured spinal cord, MCAO—middle cerebral artery occlusion, HCV—hepatitis C virus, SCIRI—spinal cord ischemia-reperfusion injury, LNP—lipid nanoparticles.
